# Application of Cell-Derived Extracellular Vesicles and Engineered Nanovesicles for Hair Growth: From Mechanisms to Therapeutics

**DOI:** 10.3389/fcell.2022.963278

**Published:** 2022-07-14

**Authors:** Prakash Gangadaran, Ramya Lakshmi Rajendran, Mi Hee Kwack, Madhan Jeyaraman, Chae Moon Hong, Young Kwan Sung, Byeong-Cheol Ahn

**Affiliations:** ^1^ Department of Nuclear Medicine, School of Medicine, Kyungpook National University, Daegu, South Korea; ^2^ BK21 FOUR KNU Convergence Educational Program of Biomedical Sciences for Creative Future Talents, Department of Biomedical Sciences, School of Medicine, Kyungpook National University, Daegu, South Korea; ^3^ Department of Immunology, School of Medicine, Kyungpook National University, Daegu, South Korea; ^4^ Department of Orthopaedics, Faculty of Medicine, Sri Lalithambigai Medical College and Hospital, Dr MGR Educational and Research Institute, Chennai, India; ^5^ Department of Biotechnology, School of Engineering and Technology, Sharda University, Noida, India; ^6^ Indian Stem Cell Study Group (ISCSG) Association, Lucknow, India; ^7^ Department of Nuclear Medicine, Kyungpook National University Hospital, Daegu, South Korea

**Keywords:** exosomes, extracellular vesicles, hair loss, hair follicles, dermal papilla, Wnt/b-catenin

## Abstract

Hair loss is one of the most common disorders that affect both male and female patients. Cell-derived nanovesicles (CDVs) are natural extracellular vesicles and engineered nanovesicles that can carry various biologicals materials such as proteins, lipids, mRNA, miRNA, and DNA. These vesicles can communicate with local or distant cells and are capable of delivering endogenous materials and exogenous drugs for regenerative therapies. Recent studies revealed that CDVs can serve as new treatment strategies for hair growth. Herein, we review current knowledge on the role of CDVs in applications to hair growth. The in-depth understanding of the mechanisms by which CDVs enable therapeutic effects for hair growth may accelerate successful clinical translation of these vesicles for treating hair loss.

## Introduction

Hair loss (alopecia) is a very common medical problem that can affect both males and females. In general, hair is a threadlike outgrowth of the outer layer of the skin (epidermis) and is a key feature of mammals, playing an important role in their appearance, social acceptance, and self-confidence (Qi and Garza, 2014). Hair loss can be caused by many factors, including hormonal changes, nutritional deficiencies, genetics, medications, inflammation, injuries, and even surgeries (Guo and Katta, 2017; Rajendran et al., 2017; Li et al., 2022). The hair follicle (HF) is a self-renewing mini-organ that undergoes a cycle of stages known as anagen, catagen, and telogen. The HF enters a non-stop cyclical process of organ regression (catagen), a relative “resting” stage (telogen) followed by a growth stage (anagen) in the hair cycle. HFs consist of components including the dermal papilla (DP), outer root sheath, hair shaft, inner root sheath, and sebaceous gland (Al-Nuaimi et al., 2010; Qi and Garza, 2014; Oh et al., 2016). The complexity of the mechanism involved in HF growth is not well understood; however, it is known that any disturbance in the HF cycle or among the range of different cells involved will lead to hair loss (Toyoshima et al., 2012).

Currently, many treatments for hair loss are commonly available, including conventional chemical approaches (minoxidil, finasteride, or herbal extracts), platelet-rich plasma, and hair transplantation (Gentile and Garcovich, 2020). Among these, only autologous follicular unit transplantation is a reliable surgical option, although the number of donor follicles is often limited. Moreover, none of these treatment methods can individually produce bring satisfactory results and they can have unpredictable outcomes or are associated with significant adverse reactions and do not lead to a permanent cure (Wagner and Kenreigh, 2007; Suchonwanit et al., 2018; Taghiabadi et al., 2020). This has led researchers to explore alternative therapies for hair restoration in alopecia.

Current advancements in cell culture methodology, tissue engineering techniques, and surgical equipment have given new hope for treating hair loss (Taghiabadi et al., 2020). Cell-based therapies in hair growth can include cells that produce factors inducing hair growth or the products of these cells can be isolated and used. These techniques are less expensive compared with the conventional therapies mentioned above. Many researchers have cultured and studied DP cells (DPCs) to use for hair growth or generation of HFs. Researchers have shown that a hair structure can be created from stem cells and mice DPCs; however, this was unsuccessful in humans because of the loss of trichogenic ability and the poor functionality of the cultured human DPCs (Ji et al., 2021; Llamas-Molina et al., 2022).

Mesenchymal stem cells (MSCs) are capable of self-renewal and differentiation into various mesenchymal tissues (osteogenic, adipogenic, and chondrogenic cell lineages) and have been identified as a promising cell-based therapy for regenerative medicine (Han et al., 2019; Shammaa et al., 2020). Several research groups have studied the stem cell therapies for treating hair loss including adipose-derived stem cells (ADSCs) (Fukuoka et al., 2017), umbilical cord blood-derived MSCs (Oh et al., 2020), and human HF MSCs (Ou et al., 2020). Though these cell-based therapies have shown promising results they have yet to achieve the desired results in hair growth. Moreover, the therapeutical effects of cells are also due to secretion factors, such as extracellular vesicles (EVs).

EVs are nano-sized biological materials released by almost all cells, including those of mammals, plants, and microorganisms (Woith et al., 2019; Herrmann et al., 2021). EVs are classified into small EVs (exosomes), macrovesicles, apoptotic bodies, and larger vesicles. In recent years, the therapeutic potential of different sources of EVs have been used in regenerative medicine and drug delivery (Gangadaran and Ahn, 2020; Romano et al., 2020; Gangadaran et al., 2021; Oh et al., 2021). With advancing understanding of the isolation methodology of EVs, researchers have developed EV mimetics that have been engineered for practical clinical application, making them an alternative therapeutic biomaterial in translation medicine. Recently, studies have been conducted using cell-derived nanovesicles (CDVs) on hair growth *in vitro* and on human HFs *in vivo*. This review presents an outline of hair loss, EVs, and the function of CDVs and engineered nanovesicles (eNVs) that can be used as a therapeutic arm in hair regeneration. We discuss current progress in promising research focused on CDV therapy for hair regeneration, including their therapeutic assessments and highlight the gaps that limit its emergence as a biologic product.

## Cell-Derived Nanovesicles

### Extracellular Vesicles

**FIGURE 1:**
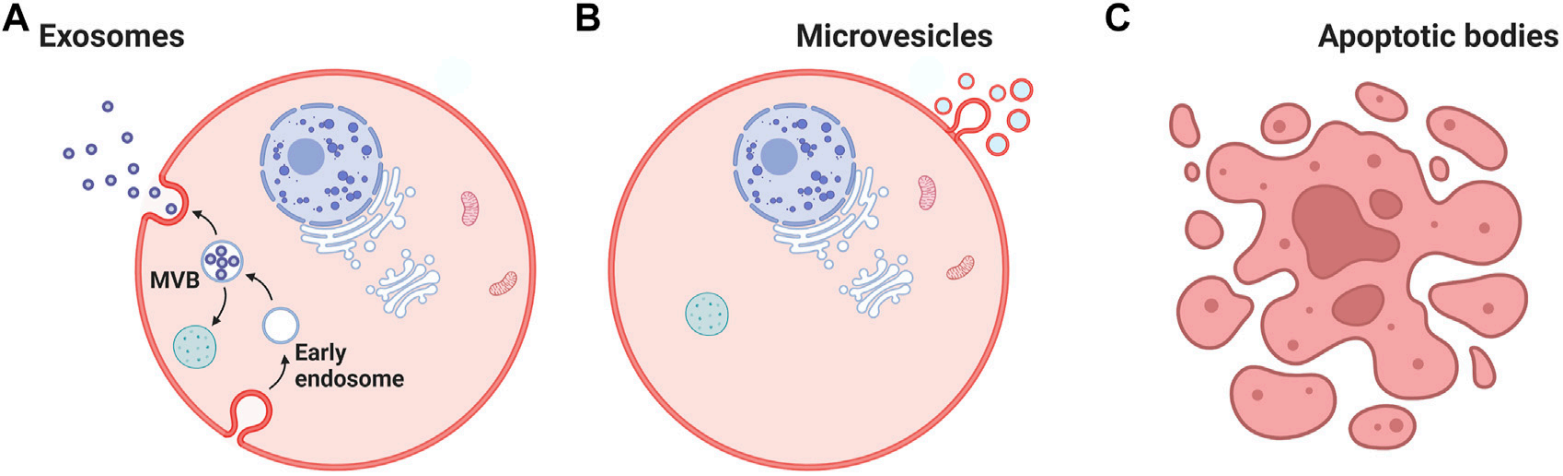
Cell-derived nanovesicles. **(A)** Biogenesis of extracellular vesicles. **(B)** Cargoes (Contents) of extracellular vesicles, such as lipids, proteins, miRNA, mRNA, and DNA. **(C)** Apoptotic bodies. MVB: multi-vesicular bodies. Created with BioRender.com.

All cells are known to release a large variety of membrane vesicles such as exosomes and microvesicles into the extracellular milieu and cell culture media (Johnstone et al., 1987; Alenquer and Amorim, 2015). The term exosome or small EVs refers to a membrane vesicle with a diameter of 30–150 nm (Doyle and Wang, 2019). Exosome term was coined by Dr. Rose Johnstone while understanding the biologic process that triggers the transformation of a reticulocyte to a mature erythrocyte (Théry et al., 2002); they are formed through multi-vesicular bodies (MVB), which appear along the endocytic pathway, are characterized by the presence of vesicles in their lumen (i.e., intraluminal vesicles) formed by inward budding from the limiting membrane (Gangadaran and Ahn, 2020) (Figure 1A). Intraluminal vesicle and MVB processes require induction of membrane curvature, inclusion of specific cargo, and membrane fission for release. MVB fusion with a lysosome can result in destruction of the MVB cargo; however, fusion with the plasma membrane results in the secretion of the vesicles into the extracellular space.

**FIGURE 2:**
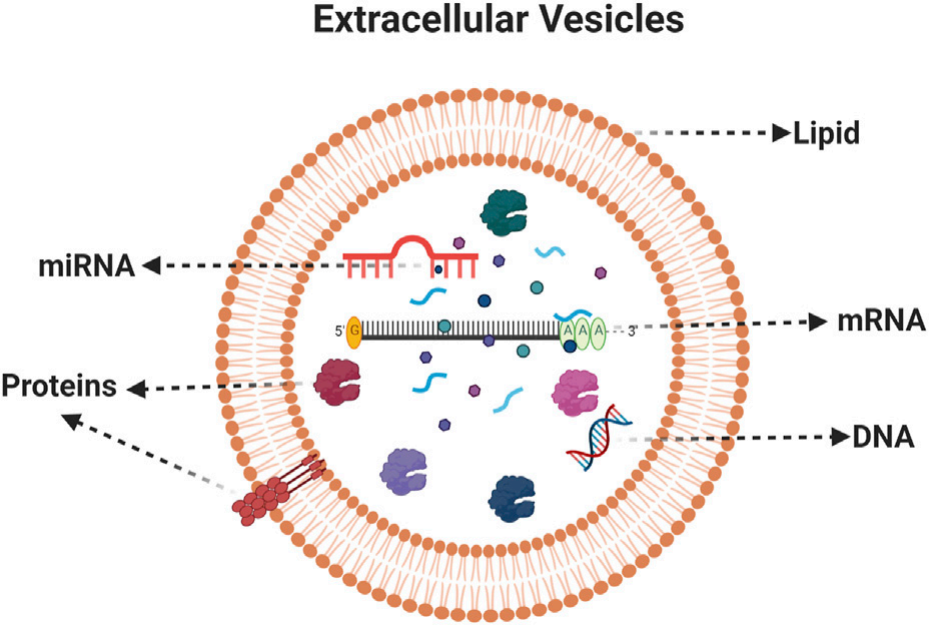
Composition of extracellular vesicles. A typical EVs consists of Lipids, proteins, mRNA, miRNA and DNA. Created with BioRender.com.

Microvesicles (100–100 nm) and apoptotic bodies (50–5,000 nm) represent a relatively heterogeneous population of vesicles that are formed by the outward budding and fission of the cell membrane (Doyle and Wang, 2019) (Figures 1B,C). Both exosomes and microvesicles characteristically carry a cargo and can deliver this efficiently to cells in remote locations. EVs were initially considered as a garbage materials or cellular dust released by the cells until studies in 1973 (Mathivanan et al., 2012). Recent information from a range of cell type reveals that EVs can contain up to 9,769 proteins, 1,116 lipids, 3,408 mRNAs, and 2,838 miRNAs (ExoCarta, 2022) (Figure 2). Importantly, the molecular content of EVs is related to their own cell type and reflect the EV biogenesis.

**FIGURE 3:**
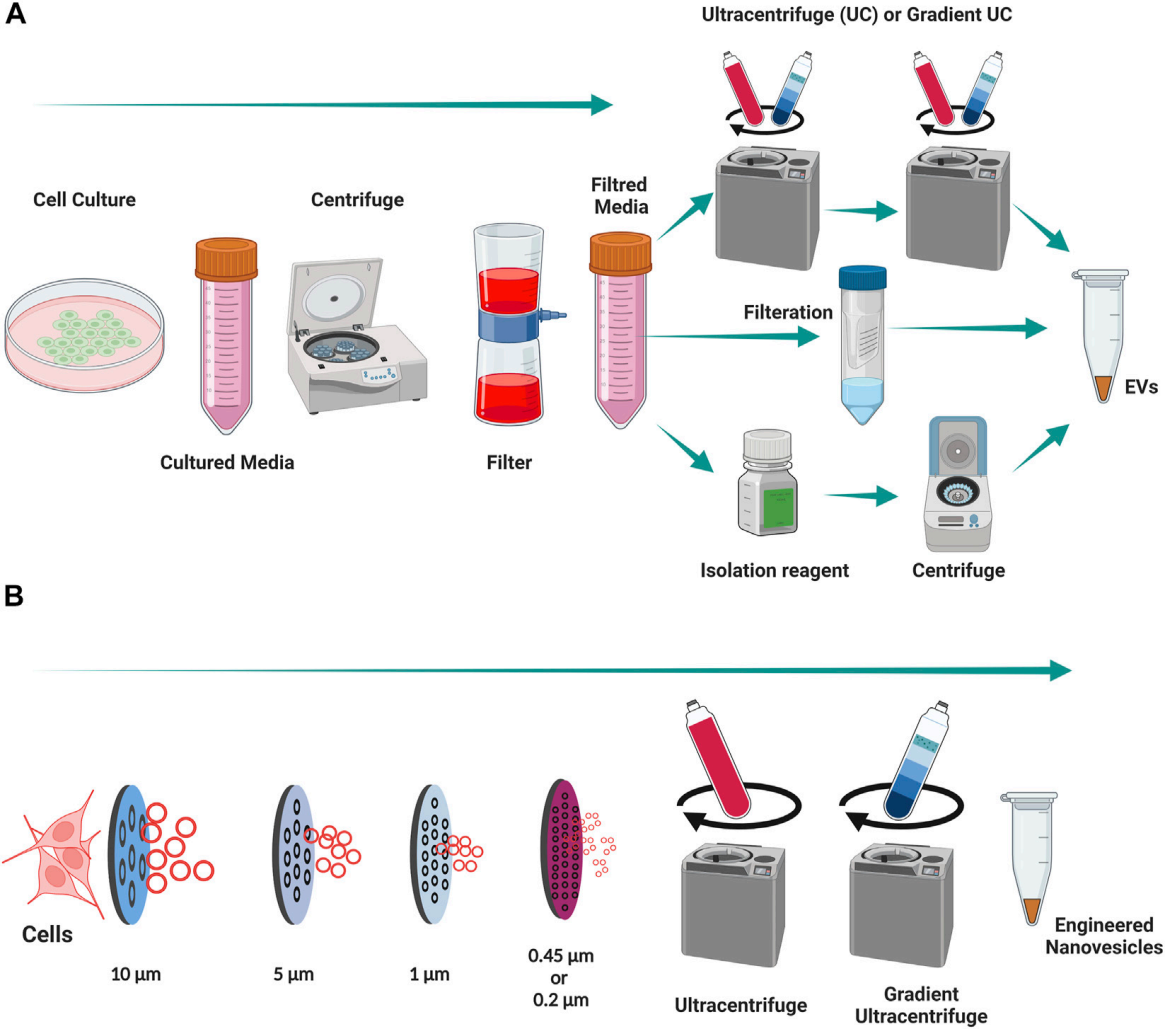
Isolation of EVs and engineering of nanovesicles from Cells. **(A)** An illustration of isolation of EVs from cell cultured media. **(B)** An illustration of engineering of nanovesicles from cells. Created with BioRender.com.

Exosomes, which originate from endolysosomal compartment are more enriched in MHC-II and tetraspanins (CD63, CD81, and Annexin A5) when compared with macrovesicles (positive marker of Annexin A1) (Jeppesen et al., 2019). EVs can hold numerous bioactive lipids, proteins, DNA, mRNAs, non-coding miRNAs, tRNAs, rRNAs, nucleolar RNAs, and long non-coding RNAs that are later transferred as messengers of intercellular communications (Malkin and Bratman, 2020; Guo et al., 2021). Cell-to-cell communication is said to the main category of communication in living organisms, but recent findings suggest that there are other ways to communicate between cells. Recent studies show those EVs represent cell-to-cell communication to distal cells in the organism (Simons and Raposo, 2009) and this is considered as a vital tool for long distance communication. This phenomenon can be used for therapeutics as this enables the delivery of functional active biomaterials into the receptor cells to modulate their behavior for beneficial effects. The EVs can be isolated by different isolation methods, mainly by ultracentrifugation, gradient ultracentrifugation, filtration and isolation reagents (Figure 3A).

### Engineered Nanovesicles

The majority of studies frequently use naturally secreted EVs as therapeutics in regenerative medicine (Romano et al., 2020). However, preclinical (small animal) and clinical research is restricted because of the low yields of natural EVs produced by cells (Gangadaran and Ahn, 2020; Rajendran et al., 2021a; Li et al., 2021). To move vesicle-based therapies into clinics, researchers must overcome this limitation on large-scale production of EVs. Recently researchers have sought to overcome this by developing a new production method for eNVs, similar to natural EVs, from cells (Jang et al., 2013). The eNVs are generally created by the extrusion of live cells though a series of micrometer or nanometer-sized membranes, followed by filtration to control the size of eNVs (Figure 3B). Previous studies suggest that with the same number of cells, approximately 20-to-100-fold more eNVs can be generated with in a twentieth of the time required for EV production (Jang et al., 2013; Li et al., 2021). The development of eNVs holds great value for translational nanomedicine.

## Cell-Derived Nanovesicles and Their Action of Mechanism for Hair Growth

**FIGURE 4:**
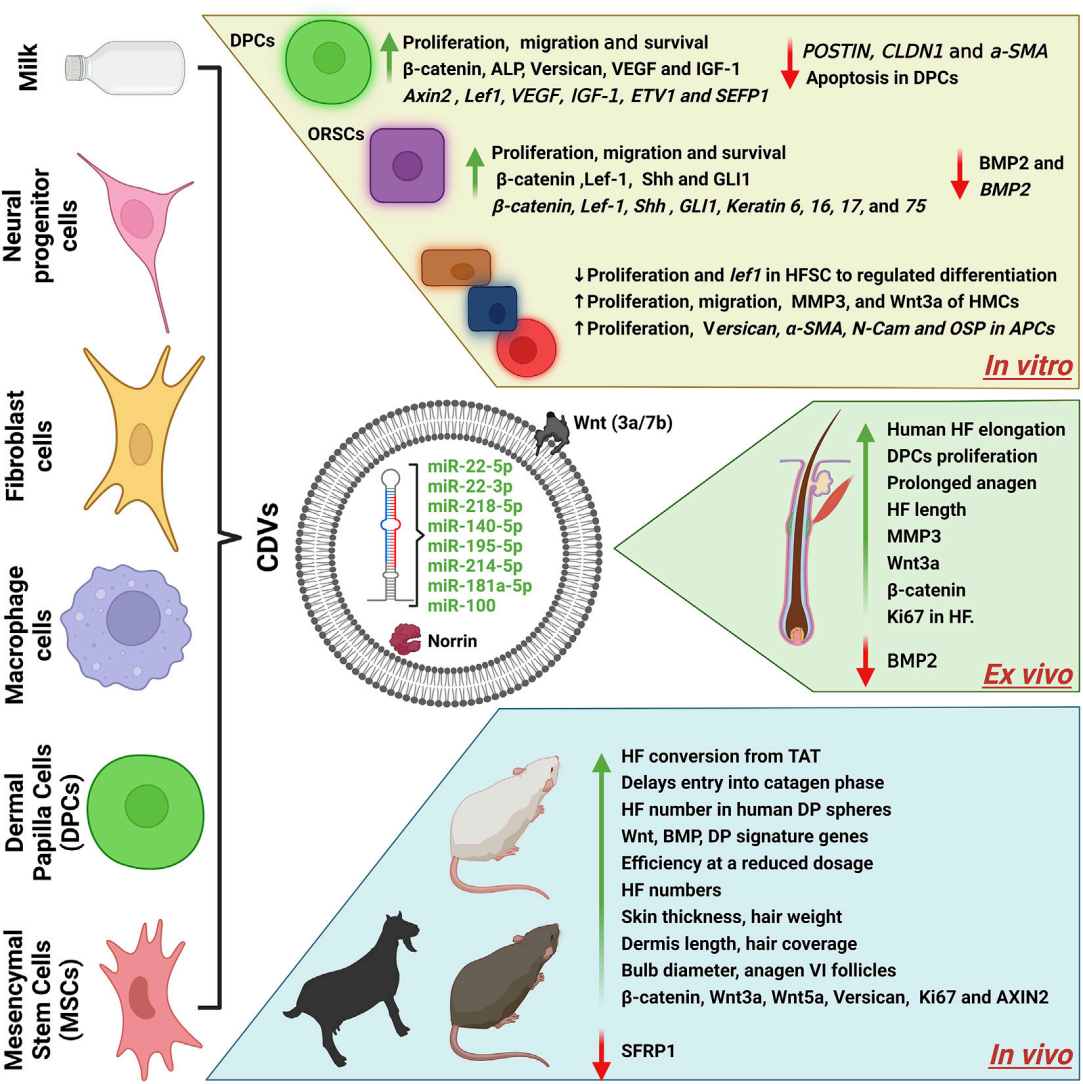
A schematics diagram of cell-derived nanovesicles for hair growth. The cargoes (miRNA/proteins) of CDVs and their effects *in vitro*, *ex vivo*, and *in vivo*. CDVs, cell-derived nanovesicles; BM-MSCs, bone marrow derived mesenchymal stem cells; DPC, dermal papillae cells; HF, hair follicles; HFSCs, hair follicle stem cells; ADSCs, adipose-derived stem cells; ORSCs, outer root sheath cells; HMC, hair matric cells; TAT, telogen to anagen transition; VEGF, vascular endothelial growth factors; IGF-1, insulin growth factors; Shh, sonic hedgehog; Lef-1, lymphoid enhancer binding factor 1; ETV1, ETS variant transcription factor 1; α-SMA, alpha-smooth muscle actin; MMP3, matrix metallopeptidase 3; Wnt3a, Wnt family member 3A; BMP2, bone morphogenetic protein 2; ALP, alkaline phosphatase; AXIN2, axis inhibition protein 2; GLI1, GLI family zinc finger 1; SFRP2, secreted frizzled related protein 2. Created with BioRender.com.

In recent years, remarkable advancements have been made in understanding the biological aspects of natural EVs and very recently also in eNVs. This review is focused on the role of CDVs (EVs and eNVs) in hair growth therapeutics as drug delivery vehicles (Figure 4; Table 1). EVs are regarded as a new player in cell-to-cell communication. The natural release of EVs from cells into the extracellular environment where they can interact with other cells and effect on biology of the recipient cells. Released EVs in cell culture media enable their isolation and subsequent use as therapeutic agents (Woith et al., 2019; Romano et al., 2020). Both EVs and eNVs exert their therapeutical effects by interacting with the recipient cells or by releasing their cargoes (e.g., miRNA and proteins) after internalization.

**TABLE 1:** Cell-derived nanovesicles in promotion of hair growth.

Type of CNVs	CDVs source	Isolation Methods	Modification	Cargoes	Outcome (*In vitro or ex vivo*)	*In vivo* (delivery route)	Outcome (*In vivo*)	References
EVs	Mouse BM-MSCs	UC	Naïve EVs	Unknown	↑Proliferation, ↑ Migration, ↑Survival of DPCs and ↑ expression (mRNA) and secretion of VEGF and IGF-1 from DPCs	C57BL/6 Mice, (I.D)	↑ HF conversion from TAT, ↑ Wnt3a, ↑ Wnt5a, and ↑Versican	Rajendran et al., (2017)
Exo	Human DPCs	UC	Naïve Exo	Unknown	↑Proliferation, ↑Migration ↑Survival, ↑Shh, ↑β-catenin of ORSCs	C57BL/6 Mice (I.D)	↑ HF conversion from TAT and delays entry into catagen phase	Zhou et al., (2018)
Exo	Human DPCs	Isolation reagent	Naïve Exo	Unknown	↑Human HF Elongation, ↑DPCs Proliferation in HFs	C57BL/6 Mice (Co-implant)	↑ HF no in human DP spheres, ↑ Wnt, BMP, DP signature genes	Kwack et al., (2019)
Exo	Human BM-MSCs	Isolation reagent	Naïve Exo	Unknown, Loaded with UK5099	-	C57BL/6 Mice (Microneedle, S.C and topical (UK5099)	↑Treatment efficiency at a reduced dosage	Yang et al., (2019)
Exo	SWC goat DPCs	UC	Naïve Exo	miR-22-5p	↓ Proliferation and Targets *lef1* in HFSC to regulated Differentiation	-	-	Yan et al., (2019)
EVs	Human Dermal Fibroblast	UC	Stimulated with bFGF and PDGF-AA	Norrin	↑ Human HF Elongation, ↑Proliferation of DPCs, Norrin-encoding genes (*↑ETV1, ↑SEFP1, ↓ POSTIN, ↓CLDN1* and *↓a-SMA*) of DPCs	-	-	Riche et al., (2019)
EVs	Human DPCs	UC	Naïve EVs with or without OSA hydrogel	Unknown	↑Proliferation, ↑Migration, ↑ MMP3, ↑ and Wnt3a of hair matrix cells. Prolonged anagen phase, ↑ length, MMP3, ↑ Wnt3a, ↑β-catenin, ↓BMP2 of cultured HFs	C57BL/6 Mice, (S.C)	↑ HFs, ↑ Skin thickness and ↑ Ki67	Chen et al., (2020a)
EVs	Mouse macrophage	UC	Naïve EVs	Wnt3a & Wnt7b	↑Proliferation, ↑Migration ↑Survival, ↑β-catenin, ↑ALP, ↑Versican, *Axin2* and *Lef1* in DPCs. ↑ Human HF Elongation	BALB/c mice (I.D)	↑ hair weight, ↑ hair numbers and ↑ dermis length	Rajendran et al., (2020)
EVs	Mouse DPCs	Filtration	3D cultured EVs with or without keratins	miR-218-5p	-	C57BL/6 Mice, (S.C)	↑ hair coverage, ↑β-catenin and *↓* SFRP2	Hu et al., (2020)
sEVs	Human DPCs	UC	Low passage Naïve EVs	miR-140-5p	↑ Elongation, ↑ remaining in anagen and ↑ Ki67 in HFs. ↑Proliferation, both gene and proteins of ↑β-catenin, ↑Lef-1, ↓BMP2, ↑Shh and ↑GLI1 in ORSCs	-	-	Chen et al., (2020b)
CDVs	Human neural progenitor cell	Density-Gradient UC	Engineered CDVs	miR-100	↑β-catenin, ↑C-myc and ↑Cyclin D1 in DPCs. ↑ Ki67 in HFs	C57BL/6 Mice, (S.C)	↑Skin thickness, ↑Bulb diameter and ↑anagen VI follicles and ↑β-catenin	Cao et al., (2021)
EVs	Human Fibroblast	UC	Naïve EVs	Wnt3a	↑Proliferation, ↑β-catenin, *Axin2* and *Lef1* in DPCs. ↑Proliferation, ↑Migration, ↑*Keratin 6, 16, 17, 75* in ORSCs. ↑ Human HF Elongation	-	-	Rajendran et al., (2021b)
EMs	Mouse macrophage	Density-Gradient UC	Engineered EMs	-	↑Proliferation, ↑β-catenin, ↑ALP, ↑Versican, ↑Survival, *Axin2* and *Lef1* in DPCs. ↑ Human HF Elongation	C57BL/6 Mice, (I.D)	↑ Dermis length	Rajendran et al., (2021a)
EVs	Mouse DPCs	UC	Naïve EVs	miR-195-5p, miR-214-5p and miR-218-5p	↑Proliferation, ↑ Versican, α-SMA, N-Cam and Osteopontin in ADSCs	-	-	Kazi et al., (2022)
Exo	Mouse ADSCs	Isolation reagent	Naïve Exos	miR-22-5p and miR-22-3p	↑Proliferation, ↑Migration and *↓* Apoptosis in DPCs	C57BL/6 Mice, (S.C)	↑ HF, ↑ Skin thickness. ↑ Hair coverage, ↑*Wnt3A* and *AXIN2.* ↓ *SFRP1*	Li et al., (2022)
Exo	Angora rabbit DPCs	Isolation reagent	Naïve Exos	miR-181a-5p	↓ *WIF1,* ↑Proliferation and ↓Apoptosis in HFSCs. ↑ Rabbit HF Elongation	-	-	Zhao et al., (2022)
Exo	Colostrum Milk	UC	Naïve Exos	-	↑Proliferation and ↑β-catenin expression in DPCs	C57BL/6 Mice, (S.C)	↑HF number, ↑ ki67, ↑ Wnt3a and ↑ β-catenin	Kim et al., (2022)

EVs, extracellular vesicles; Exo, exosomes; CDVs, cell-derived vesicles; EMs, extracellular vesicles mimetics; BM-MSCs, bone marrow-derived mesenchymal stem cells; UC, ultracentrifuge; DPC, dermal papillae cells; HF, hair follicles; HFSCs, hair follicle stem cells; ADSCs, adipose-derived stem cells; SWC goat, Shaanbei White cashmere goat; TAT, telogen to anagen transition; VEGF, vascular endothelial growth factors; IGF-1, insulin growth factors; bFGF, basal fibroblast growth factor; PDGF-AA, platelet-derived growth factor-AA (two A subunits); Shh, Sonic hedgehog; Lef-1, lymphoid enhancer binding factor 1; ETV1, ETS variant transcription factor 1; α-SMA, alpha-smooth muscle actin; MMP3, matrix metallopeptidase 3; Wnt3a, Wnt family member 3A; BMP2, bone morphogenetic protein 2; ALP, alkaline phosphatase; AXIN2, axis inhibition protein 2; GLI1, GLI, family zinc finger 1; SFRP2, secreted frizzled related protein 2; UK5099, small molecular drug; OSA, oxidized sodium alginate; I.D, intradermal; S.C, subcutaneous.

### Dermal Papilla Cells Derived Extracellular Vesicles

Human DPC-derived exosomes (DPC-Exos) treatment of C57BL/6 mice was shown to increase the skin thickness, bulb diameter, hair cycle score, and percentage of HFs. The time course experiment for anagen-to-catagen transition analysis showed an increase in skin thickness, bulb diameter, and HFs in anagen (%) in DPC-Exos-treated mice. Moreover, this study also showed that DPC-Exos treatment increased β-catenin and Shh expression in mouse skin tissues. DPC-Exos increased the proliferation and migration of outer root sheath cells (ORSCs). Additionally, DPC-Exos treatment promoted S- and S/G1-phase entry of ORSCs and increased the expression of both mRNA and protein levels of β-catenin and Shh in ORSCs. These results suggest that DPC-Exos could induce hair growth by activating the β-catenin and Shh signaling pathways (Zhou et al., 2018). Another study investigated the hair growth capacity in both two-dimensional (2D) and three-dimensional (3D) cultured human DPC-Exos (2D or 3D DPC-Exos) on cultured human HFs and in C57BL/6 mice. Treatment of 3D DPC-Exos increased the proliferation of DPCs and ORSCs and increased the expression of several growth factors (IGF-1, KGF, and HGF) in DPCs. Treatment using 3D DPC-Exos of cultured human HFs increased the hair shaft elongation. The local injections (skin) of 3D DPC-Exos into C57BL/6 mice induced TAT and also increased the retention of anagen in mice. Treatment with 3D DPC-Exos in DP spheres augmented HF neogenesis through the increase Wnt, BMP and DP signature genes. Similar results were also observed with 2D DPC-Exos treatments. These results suggested that DPC-Exos induced hair growth *in vitro* and *in vivo* regardless of DPC culture conditions (Kwack et al., 2019). The effects of exosomal miRNA derived from Shaanbei White cashmere goat on proliferation and differentiation of DPCs and HFSC. The study investigated the miRNAs present in DPC-Exos by miRNA high-throughput sequencing and found that 111 miRNAs were differentially expressed between DPC-Exos and DPCs to a significant degree. Out of 111 miRNAs, 34 found to be involved with regulating HFSC proliferation and differentiation; miR-22-5p was expressed the lowest in DPC-Exos compared with expression in DPCs. The overexpression of miR-22-5p in HFSCs reduced their proliferation and targeted expression of the *LEF1* gene by downregulation. This study revealed that miR-22-5p was a major player in regulation of HFSC proliferation and differentiation (Yan et al., 2019). The sustained release of DPC-derived EVs (DPC-EVs) from injectable microgel (oxidized sodium alginate, OSA) supports hair growth. DPC-EV treatment increased the cell proliferation and migration in hair matrix cells. DPC-EVs mixed with an OSA-hydrogel protected DPC-EVs from degradation and permitted their sustained release, which increased the expression (mRNA and protein) levels of MMP3 and Wnt3a of hair matrix cells. Furthermore OSA-DPC-EVs treatment increased the prolongation of anagen phase and HF length in cultured HFs. OSA-DPC-EVs increased the HF in mice and increased the expression of MMP3, Wnt3a, and β-catenin, deceased the expression of BMP2 in cultured HFs and mice. This study results showed that improved delivery method for the prolonged delivery of therapeutic EVs (Chen et al., 2020a). The therapeutical effects of 2D and 3D culture conditions on mouse dermal DPCs (2D DPCs or 3D spheroids) and their exosomes (2D DPC-EXOs or 3D DPC-EXOs) on hair regeneration were studied. First, the authors subcutaneously injected the DiD (a lipophilic dye; 1,1-Dioctadecyl-3,3,3,3-tetramethylindodicarbocyanine)-labeled 2D DPCs or 3D DPCs with or without keratins into the dorsal skin of C57BL/6 mice; this demonstrated that 3D DPCs persisted longer in the presence of keratins at the injection site. Treatment with 2D DPCs or 3D DPCs with keratins showed that 3D DPCs increased the expression of β-catenin and CD133in Ki67-positive cells more than that in 2D DPCs with keratin treatment. 3D DPC treatment increased the expression of β-catenin and pERK and decreased that of the SFRP2. The therapeutical effects of these cells were based on secretory factors, including EVs, and therefore these were isolated from 2D and 3D DPCs. Analysis of miRNA revealed that expression of miR-218-5p was upregulated in 3D DPC-EXOs in comparison with that in 2D DPC-EXOs. Furthermore, antibody array results showed that basic fibroblast growth factor (bFGF) was enriched in 3D DPC-EXOs compared with that in 2D DPC-EXOs. Treatment of mice with 3D DPC-EXOs showed that these increased hair coverage area and expression of β-catenin but decreased expression of SFRP2; inhibition of miR-218-5p expression abolished hair growth effects. This study effectively revealed that miR-218-5p regulated HF development by downregulating expression of the WNT signaling inhibitor SFRP2 (Hu et al., 2020). Another study investigated therapeutical effects of human DPCs derived small EVs (sEV) on ORSCs and hair matrix cells. The authors tested the effects of sEVs from passage 3 (P3) and 8 (P8) of DPCs and showed that DPC-EV (P3) increased shaft elongation, prolonged anagen stage, and increased Ki67 expression in HFs compared with effects induced with DPC-EV (P8). DPC-EV Treatment with P3 increased the cell proliferation and expression of both genes and proteins of β-catenin, LEF-1, Shh, and GLI1 while decreasing expression of BMP2 in ORSCs. miR-140-5p was enriched in DPC-EV (P3) compared with that in DPC-EV (P8). Further experiments showed that miR-140-5p targeted BMP2 expression; treatment with an miR-140-5p inhibitor abolished hair growth effects. This study showed that miR-140-5p from low passage number DPC-EVs impacts HF growth and hair cycling (Chen et al., 2020b). EVs derived from DPCs (DPC-EVs) on hair inductive gene expression in adipose derived stem cells (ADSCs)was investigated. The expression of hair inducing miRNAs (miR-195-5p, miR-214-5p, and miR-218-5p) in DPC-EVs was demonstrated. DPC-EVs treatment increased cell proliferation, expression of versican and osteopontin mRNA as well as that of *α-SMA* and *N-Cam* in ADSCs. The DPC-EVs treated ASP cells showed the similar characteristics of DPCs (Kazi et al., 2022). A recent study used Angora rabbit DPCs-derived exosomes (DPC-Exos) for hair growth. The authors showed that miR-181a-5p was enriched in EVs of P1 than P8. miR-181a-5p targets *WIF1*, and DPC-Exos treatment increased cell proliferation and decreased the apoptosis of HFSCs. The treatment of DPC-Exos increased shaft length of Angora rabbit HFs *ex vivo* (Zhao et al., 2022)*.*

### Stem Cells Derived Extracellular Vesicles

The role and function of mouse bone marrow-derived MSCs derived EVs (MSC-EVs) on hair growth was investigated. The study then showed that internalization of MSC-EVs into DPCs increased the proliferation and migration of DPCs through activating or elevating the levels of phosphorylated AKT (pAKT), pERK, and Bcl-2. Moreover, MSC-EVs *in vitro* treatment of DPCs increased the expression (mRNA) and secretion (protein) of VEGF and IGF-1. The intradermal injection of MSC-EVs at multiple sites increased hair growth in C57BL/6 mice and further confirmed the dermis length (anagen from telogen: TAT) via histological analysis. Additionally, MSC-EV treatment increased the expression of Wnt3a, Wnt5, and versican in treated skin. This study results showed that MSC-Evs treatments can be a candidate for hair growth (Rajendran et al., 2017). A study was performed to elucidate change of Exos’s residence time by their administration routes. MSC-Exos were administered to mice through a microneedle patch or by subcutaneous (S.C) injection. The imaging results showed that DiD-labeled MSC-Exos were retained longer when delivered by microneedle patch than by S.C delivery. A microneedle patch delivery of UK5099, MSC-Exo, or MSC-Exo with UK5099 was performed on mice and showed that MSC-Exo or UK5099 treatment increased the anagen stage of HF by the 18th day, but the combination treatment produced an increase in the anagen stage within 12 days. Similarly, sole treatment with MSC-Exo or UK5099 increased the hair-covered area, HF anagen percentage, and hair density while a combined treatment showed the highest effects. They also achieved treatment efficiency at a reduced dosage in microneedle patch delivery compared with that achieved using S.C (MSC-Exo) or topical (UK5099) treatments. These results suggested that MSC-Exos could act as an anagen inducer and that this can be achieved at low dose with microneedle patch delivery (Yang et al., 2019). The effect of exosomes derived from mouse ADSCs (ADSC-Exos) on immune-mediated alopecia was investigated. ADSC-Exos treatment promoted DPC proliferation and migration and inhibited apoptosis *in vitro*. ADSC-Exos treatment (S.C injection) to C57BL/6 mice promoted HF and skin thickness and hair coverage. miRNA analysis revealed that miR-22-5p and miR-22-3p were enriched in ADSC-Exos in comparison to expression in DPCs. ADSC-Exos treated *in vivo* skin showed increased expression of *Wnt3A, AXIN2,* and *SFRP1* mRNA. This study provides the potential for developing ADSC-Exos as promising therapeutic EVs for immune-mediated alopecia (Li et al., 2022).

### Macrophages Derived Cell-Derived Nanovesicles

Effect of mouse macrophage-derived EVs (MAC-EVs) on hair growth was tested *in vitro*, cultured HFs, and *in vivo*. The study showed that MAC-EVs contained Wnt3a and Wnt7b at the EV membrane. MAC-EVs treatment increased proliferation and migration of DPCs through activating or elevating the levels of phosphorylated AKT (pAKT), pERK and Bcl-2. Furthermore, MAC-EVs treatments increased the levels of β-catenin, ALP, and versican protein and *Axin2* and *Lef1* mRNA in DPCs. The intradermal injection of MAC-EVs at multiple sites increased hair growth in BALB/c mice, which was confirmed by hair weight and histology of the HF number and TAT dermis length. MAC-EVs treatment of cultured human HFs increased the hair shaft elongation. These results showed that MAC-EVs containing Wnt proteins on the membrane of EVs can activate the Wnt/β-catenin signaling cascade to induce hair growth (Rajendran et al., 2020). EV mimetics from macrophage cells (MAC-EMs) effect on hair growth was studied both *in vitro* cell culture and *in vivo* animal model. Authors engineered the MAC-EMs through extrusion of macrophage through 10-, 5-, and 1-μM polycarbonate membrane filters and a 0.45-μM filter. MAC-EMs were concentrated and purified using an ultracentrifugation and density gradient ultracentrifuge. Treatment with MAC-EMs resulted in cell proliferation and expression of β-catenin, PCNA, versican, ALP, pAKT, pERK, and BCL-2 in DPCs *in vitro*. Treatment of C57BL/6 mice with MAC-EMs increased the dermis thickness and pigmentation of mouse skin. Finally, treatment of MAC-EMs increased the shaft elongation of human HFs *ex vivo*. This study suggest that MAC-EMs also possess hair growth therapeutical effects as do natural MAC-EVs; therefore, EMs can be engineered in larger scale than natural EVs and have similar therapeutical effects (Rajendran et al., 2021a).

### Fibroblast Derived Extracellular Vesicles

Effect of the dermal fibroblast (DFs) derived EVs with or without bFGF and PDGF-AA stimulation (st-EVs) on HF growth was experimented. The human HFs treated with st-EVs showed increased elongation of the hair shaft compared with that found using ctrl-EVs or control (no treatment). st-EVs increased the proliferation of DPCs and activated secretion of Wnt/β-catenin ligand in DPCs. Further results suggested that st-EVs contain Norrin, and this may activate the Norrin-specific receptor Frizzled4. st-EVs treatment regulated expression of Norrin-encoding genes (increased *ETV1* and *SEFP1* expression and decreased *POSTIN*, *CLDN1*, and *a-SMA* expression) in DPCs. Thus, Norrin may play a role in HF physiopathology (Riche et al., 2019). Another studied used human normal fibroblast-derived EVs (hFB-EVs) for hair growth using DPCs and ORSC and examined the molecular mechanisms responsible for hair growth in HFs. The authors showed that Wnt3a was more enriched in EVs compared with that present in cells, and more than 75% of hFB-EVs contained Wnt3a in the EV membrane. hFB-EV treatment increased cell proliferation and expression of β-catenin and *Axin2* and *Lef1* mRNA in DPCs. In addition, treatment also increased the cell proliferation and migration and expression of keratin 6, 16, 17, and 75 in ORSCs. Finally, treatment of hFB-EVs increased the shaft elongation of human HFs *ex vivo* (Rajendran et al., 2021b).

### Neural Progenitor Cells Derived Cell-Derived Nanovesicles

Human neural progenitor cells (ReNcells) engineered cell-derived nanovesicles (ReN-NVs) for hair growth effects was investigated. ReN-NVs were produced through serially extrusion via membrane filters with pore sizes of 1, 0.4, and 0.2 μM and purified by density-gradient ultracentrifugation. ReN-NV treatment increased cell proliferation and expression of β-catenin, C-MYC, and Cyclin D1 in DPCs. ReN-NV treatment also increased Ki67 expression in cultured human HFs. ReN-NVs treatment increased skin thickness, bulb diameter, anagen VI follicles, and β-catenin expression in mice. miRNA profiling analysis revealed that miR-100 was much more abundant in ReN-NVs than in NVs derived from control HEK293 cells. Furthermore, miR-100 inhibition verified its key role in ReN-NV-induced β-catenin signaling activation. This study provides the potential for large-scale production of NVs for hair growth therapies (Cao et al., 2021).

### Milk Derived Extracellular Vesicles

Recent study used an isolated EVs from colostrum (first milk) and investigated their therapeutical effects on hair growth. Milk-EVs treatments increased the proliferation and β-catenin expression in DPCs. *In vivo* treatment of milk-EVs to mice increased the hair regeneration, HF number, expression of ki67, Wnt3a and β-catenin. They also showed that milk-EVs can be freeze dried and used for hair growth again without affecting therapeutical effects on *in vitro* and *in vivo* (Kim et al., 2022).

We reviewed seventeen studies in this review, out of it, eight studies have used DPCs for their CDV source, followed by three studies that used stem cells, followed by macrophages, fibroblasts, while two studies used neural progenitor cells or milk (Table 1). Approximately 75% of the studies described above show activation of the Wnt/β-catenin-relayed signaling pathways. It is well known that Wnt1a, Wnt3a, Wnt7a, and Wnt7b are inducers of hair growth because of their ability to activate the Wnt/β-catenin signaling in DPCs (Kishimoto et al., 2000). Surprisingly, many miRNAs (Table 1) have been identified that directly or indirectly activate the β-catenin signaling *in vitro* or *in vivo*. Additionally, many other unknown factors may have played a role in induction of hair growth.

## The Mechanisms of Action of Extracellular Vesicles Cargoes in Hair Growth

EVs are regarded as a new player in cell-to-cell communication. The release of EVs from cells into the extracellular environment, where they can interact with other cells to effect physiological changes, or into cell culture media, enables their isolation and subsequent use as therapeutic agents (Woith et al., 2019; Romano et al., 2020). EVs or eNVs exert their therapeutical effects by interacting with the recipient cells or by releasing their cargoes (e.g., miRNA and proteins) after internalization.

DPC-derived EVs showed that various cargoes can activate signaling pathways to induce hair growth *in vitro*, *ex vivo*, and *in vivo*. Initially, a number of studies using DPC-derived EVs demonstrated that unknown cargo factors may have played a role in induction of hair growth; for instance, human DPC-derived EVs increased/activated Shh and β-catenin expression in ORSCs *in vitro* (Zhou et al., 2018) and that of Wnt, BMP, and DP signature genes (Kwack et al., 2019) in mice. Another study showed increased expression and activation of Wnt3a and MMP3 of hair matrix cells and HFs, whereas levels of BMP2 decreased and those of β-catenin increased in HFs. Four studies with DPC-derived EVs showed that special cargoes were involved in induction of hair growth. One study used exosomes derived from Shaanbei White cashmere goat DPCs that contained miR-22-5p, which directly targets/downregulates *Lef1* mRNA to regulate cell differentiation (Yan et al., 2019). Another study showed that mouse DPC-derived EVs, and especially 3D-cultured DPC-derived EVs, carry high levels of miR-218-5p and enriched levels of bFGF to directly act on SFRP2 by decreasing its expression via activation of Wnt/β-catenin relayed signaling cascades and subsequent downstream targeting of β-catenin. bFGF present in EVs activates the FGFR downstream pathway by activating MEK and ERK1/2 and targeting bFGF downstream in DPCs (Hu et al., 2020). EVs from human DPCs were shown to contain miR-140-5p that directly targeted BMP2 by downregulating its expression and subsequently increasing the transcription and translation of β-catenin, Lef-1, SHH, and GLI1 in ORSCs (Chen et al., 2020b). Finally, another study showed that mouse DPC-derived EVs carry hair growth-related miRNAs such as miR-195-5p, miR-214-5p, and miR-218-5p and that they increased the expression of versican, α-SMA, N-Cam, and osteopontin in APCs.

Bone marrow MSC-derived EVs studies have not identified specific cargoes in the EV compartments, although these have shown hair growth induction through activation of Wnt3a, Wnt5a, and versican (Rajendran et al., 2017). Macrophage-derived EVs contain Wnt3a and Wnt7b that are located in the EV membrane, which favors the activation of Wnt/β-catenin signaling pathways. This study showed that Wnt proteins activated the frizzled receptors in the DPC plasma membrane and increased the β-catenin level in the cytoplasm, which then translocated to the nucleolus to activate *Axin2* and *Lef* expression (Rajendran et al., 2020).

Macrophage-derived EVs and EMs have both been shown to increase the levels of β-catenin, ALP, versican, and survival markers (pAKT, pERK, and Bcl-2) in DPCs (Rajendran et al., 2020; Rajendran et al., 2021a). Similarly, fibroblast-derived EVs carry Wnt3a or Norrin that activate the Wnt/β-catenin-relayed signaling pathways in DPCs (Riche et al., 2019; Rajendran et al., 2021b). In addition, fibroblast-derived EVs induced ORSC differentiation by increasing the transcriptional expression of keratins (Rajendran et al., 2021b), whereas activated fibroblast-derived EVs regulated the Norrin-encoding genes of DPCs (Riche et al., 2019). Another study showed that human neural progenitor cell**-**derived EVs containing miR-100-5p increased the levels of β-catenin, C-myc, and Cyclin D1 in DPCs and these effects were abolished when miR-the DPCs were treated with miR-100-5p inhibitors (Cao et al., 2021). Finally, mouse ADSC-derived EVs were shown to carry low levels of miR-22-5p and miR-22-3p; use of inhibitors against miR-22 diminished the hair inductive ability (Li et al., 2022). These results suggest that miR-100-5p, miR-22-5p, and miR-22-3p have a critical role in activating β-catenin-relayed signaling pathways.

## Advantages and Disadvantages of Cell-Derived Nanovesicles for Hair Growth

The cell source of EVs or eNVs is the most important factor that contributes to the therapeutic effect of their CDVs, as their cargos reflect their cell source. As mentioned above many studies used DPCs as the cell source for EVs for induction of hair growth, although cultured DPCs have shown a loss of trichogenic ability and weak functionality (Taghiabadi et al., 2020). The loss of a cells loses defining characteristics is reflected in their EVs as well. This was demonstrated by Chen et al. described earlier where the ability of EVs from low passage DPCs to stimulate hair growth in ORSCs and HMCs was lost at higher passage numbers (Chen et al., 2020b).

Exosomes derived from cultures of 3D human DPCs have shown similar hair inductive effects to those derived from 2D-cultured human DPCs in both *in vitro* and *in vivo*, whereas exosomes derived from mouse 3D DPC cultures exhibited significantly better hair inductive effect than those derived from 2D-cultured human DPCs. It is not clear whether the origin of cells (mouse/human) has any influence on EV contents from 2D and 3D cultures; the method of isolation also varied between these studies (Kwack et al., 2019; Hu et al., 2020). The source of DPCs are HFs, which are not readily available at request, and this could be one of the main issues in using a DPCs as an EV source (Taghiabadi et al., 2020).

Although neural progenitor cells have been used as a cell source for eNVs, these would be hard to acquire from human subjects (Cao et al., 2021). Several studies have used bone marrow MSCs and ADSCs as an EV source for hair growth; both these cells are good sources for EVs for hair growth as they available at considerable scale compared with DPCs or neural progenitor cells (Rajendran et al., 2017; Gangadaran et al., 2021). Macrophages would also be a good source for EVs or eNVs as they can be isolated from blood with minimal invasiveness. Therefore, macrophages could be isolated from the same patients who require hair growth treatment (Rajendran et al., 2020; Rajendran et al., 2021a).

A large quantity of EVs needed is inevitably required to further study the therapeutical effects of hair growth by EVs from different cell sources and also to translate this therapy into clinics. Natural EVs are well known to be produced in very low amounts (Jang et al., 2013; Gangadaran and Ahn, 2020; Li et al., 2021), and alternative methods are required to scale up the production of EVs or enhance the therapeutical effects of EVs. Consequently, researchers have developed an extrusion method to directly engineer EV-like nanovesicles from cells, which can produce more eNVs than naturally secreted EVs from the same number of cells (Jang et al., 2013; Gangadaran et al., 2018).

Two studies have shown that engineered NVs from neural progenitor and macrophage cells have a hair inductive ability (Rajendran et al., 2021a; Cao et al., 2021). Further new methodologies and studies are required to tackle these issues. Another approach to making preclinical EVs for clinics is by enhancement using 3D culture or stimulation of cells to increase the hair-inductive cargoes in EVs for improved hair induction with relatively low quantity of EVs (Riche et al., 2019; Hu et al., 2020).

Therapeutic EVs are nature’s very own nanoparticles from cells, they inherently benefit from immune tolerance, stability in systems (Gupta D. et al., 2021). In addition, as therapeutic EVs can be derived from autologous cells (EVs/NVs derived from cells obtained from the same individual), so they are less likely to be rejected by immune system (Murphy et al., 2019). Generally, EVs are not recognized by the immune system as foreign materials, so EVs are able to evade immune system (Mittal et al., 2020; Ping et al., 2021).

Several reports have studied the stability of EVs in different conditions (durations, temperatures, and freezing and thawing cycles) and most cases studies have reported isolated EVs were stored at -80°C. This condition of storage showed loss of EVs, changes in the lipid integrity of EVs morphology, loss of cargoes (such as RNA and protein), number of EVs were decreased on the other hand size range increased. The −80°C storage have other limitations such as high cost and transportation (Tessier et al., 2021; Yuan et al., 2021). However, the most favorable condition for storage of isolated EVs is −80°C.

## Clinical Development and Conclusion

CDVs therapy holds a promising therapeutic potential in hair growth based on recently reported preclinical studies. However, as this area is a new field, the clinical application of CDVs for hair growth is still lagging. No studies were found when searching using the key words “extracellular vesicles,” “exosomes,” “hair loss,” and “alopecia” in the ClinicalTrials.gov database.

Recently few piolet studies have showed exosome treatments are promising for hair growth. In a study, MSCs derived exosomes were applied in 20 patients (male and female), the outcome showed that they enhanced the hair thickness and density (Exosome for hair regeneration, 2019). Innovators at the International Society of Hair Restoration Surgeons (ISHRS) World Congress in 2020 showed a promising preliminary outcome by MSCs derived exosome treatments (Gupta A. K. et al., 2021; Sadick, 2021).

The novelty of CDV-based research implies more studies will be forthcoming. Table 1 summarizes the indicated role of CDVs in HF signaling and development. Further extensive basic research on CDVs to hair growth is required to enable the exploration of clinical treatments. We urge researchers and innovators in the field to complete the missing pieces and assist in effective clinical translation of CNVs therapy for hair loss.
